# Multivariate Data Analysis to Assess Process Evolution and Systematic Root Causes Investigation in Tablet Manufacturing at an Industrial Scale—A Case Study Focused on Improving Tablet Hardness

**DOI:** 10.3390/pharmaceutics17020213

**Published:** 2025-02-07

**Authors:** Rita Mathe, Tibor Casian, Ioan Tomuta

**Affiliations:** Department of Pharmaceutical Technology and Biopharmacy, “Iuliu Hatieganu” University of Medicine and Pharmacy, 400012 Cluj-Napoca, Romania; rita.mathe@gmail.com (R.M.); tomutaioan@umfcluj.ro (I.T.)

**Keywords:** multivariate data analysis, batch modeling, root causes investigation, tablet manufacturing, active ingredient variability, hardness improvement

## Abstract

**Background/Objectives**: Only a few studies performed at industrial scale in non-simulated conditions have investigated the effect of input variability from the product’s lifecycle on product quality. The purpose of this work was to identify the root causes for the low and variable hardness of core tablets prepared using high-shear wet granulation through batch statistical modeling and to verify the short- and long-term effectiveness of the improvement actions. **Methods**: The novelty of this study is the use of multivariate methods for the complex assessment of a wide data set belonging to two proportional composition strengths, manufactured at an industrial scale, with different tablet shapes and sizes, with the aim of identifying inter-related active ingredient and process variables with the highest impact on hardness value and for defining optimal processing conditions leading to a robust product. **Results**: Four main variables affecting the output variable were identified: API particle size, nozzle type used for granulation, wet discharge, and drying intensity. These were included in an updated control strategy (3 out of 4 variables having to be within the desired ranges: API d0.5 < 45 microns; granulation nozzle that ensures liquid dispersion into droplets; gentle wet discharge and drying processes). In the case of the product studied, the newly defined process conditions could even accommodate d0.5 up to 70 microns and still ensure adequate core tablet hardness (at least 30% above the lower specification limit) for the successive film-coating step. **Conclusions**: Besides the beneficial impact of reducing the risk for out-of-specification hardness results, this study also offered the benefit of cost avoidance and yield improvement. The improvement was confirmed through the significant average hardness increase (15–20%) and between-batch variability decrease, leading to decent sigma quality levels (2.5) for the control phase batches.

## 1. Introduction

The high-shear wet granulation process is one of the most widely used size enlargement technologies in the manufacturing process flow of solid oral dosage forms, especially in the case of high drug load products, where either the flowability and compressibility of the active ingredient has to be improved, or the dissolution rate of the active ingredient has to be modulated, in order to ensure the proper bioavailability for successful therapeutics. Despite the long history of this process and the extensive research and routine operations experience, this manufacturing process step is still amongst the most complex ones and is prone to multivariate influences from the raw materials, the equipment, and the process parameters applied [1,2,3].

When it comes to process improvement opportunities, understanding the process and product specifics in the frame of particle growth and consolidation mechanisms is of high importance [4]. A well-controlled and well-understood wet granulation process leads to increased downstream performance in the tableting, film-coating, and packaging steps, adding cost optimization and higher throughput to the main objective of yielding a robust product of high quality [5,6].

Wet granulation and tableting are two pharmaceutical manufacturing processes that are paired for many products. The complexity of their interdependence with respect to product and process performance is still a big pillar for the development of new monitoring and data analysis techniques for improvement projects of both batch-wise and continuous processes. Several approaches were assessed at the laboratory or pilot scale, including compaction simulation and mathematical modeling of granule properties variability, prediction of tablet properties from granulation process parameters, use of artificial neural networks, and data fusion [5,7,8,9,10,11,12,13,14].

Industrial-scale manufacturing processes generate large amounts of process data that contain multiple interdependent variables, fluctuating in a less controlled manner than in the case of a rigorously set up Design of Experiments [15]. This makes it difficult to properly identify the variables of interest during a root cause investigation without applying a multivariate data analysis approach. Throughout the multiple correlations shown by the loading plots of Principal Component Analysis (PCA) or (Orthogonal) Partial Least Squares models ((O)PLSs), which have to be interpreted in a case-by-case manner, several causality relationships can be found, which can lead to enhanced process control [16]. The multivariate analysis techniques can be integrated very well in one of the most effective, scientific, systematic, and statistical root cause investigation and process improvement methodologies like the Six Sigma DMAIC approach (Define, Measure, Analyze, Improve, and Control) [17], which is gaining more and more interest and applicability also in the pharmaceutical industry due to the multiple challenges faced in front of the high quality and cost-reduction requirements [18].

Routine manufacturing processes are validated and some or most of the process parameters are part of the registration file, leaving just a few options for additional improvement without major revalidation or regulatory relevant change [19]. However, during the product lifecycle, due to the implementation of alternative manufacturers for raw materials or scale-up activities, there is a need for systematic continuous improvement. These opportunities come from additional control strategies based on the assessment of historical results [16], which sometimes might be coupled with actual testing of different hypotheses at the industrial scale or designed experiments at the pilot or laboratory scale [19,20,21,22,23]; however, there are still only a few studies that have been performed at the industrial scale in non-simulated conditions.

The objective of this work was to identify the root causes leading to low and variable hardness of core tablets by applying a systematic approach based on batch statistical modeling and to verify the effectiveness of the implemented improvement actions in the short and long term. Hardness values slightly below or close to the low end of the hardness specification have no impact on product efficacy, as the dissolution rate is not affected in this case but can have a significant impact on downstream processability. Increased precautions are needed in the film-coating and/or packaging process steps to maintain the integrity of low-hardness tablets. Therefore, besides the beneficial impact of reducing the risk for out-of-specification results for in-process control parameters, this study offered a second benefit of cost avoidance and yield improvement.

The novelty of this study is given by the use of multivariate methods for identifying inter-related raw materials and process variables with the highest impact on the core tablet hardness value and for defining optimal processing conditions leading to a robust product. Another element of novelty and complexity is the assessment of two different strengths with proportional compositions having significantly different tablet shapes and sizes, a variable known to influence the mechanical properties of the tablets [24]. A wide data set was used for modeling and root cause investigation purposes and was composed of active ingredient properties, processing conditions, and intermediate product characteristics from 114 batches manufactured at the industrial scale over the course of 18 months. The effectiveness of the improvement actions was verified on an additional 46 batches, which were manufactured during the next 5 months in optimal conditions, which were identified through statistical modeling.

## 2. Materials and Methods

### 2.1. Product Summary

For this study, a high-dose immediate-release solid oral dosage form was chosen. This product is present on the market in two different declared contents/unit doses. The two strengths of the product are dose-proportional, having identical qualitative and quantitative composition, and the declared content per unit achieved by setting different targets for tablet weight, leading to differences in size and weight (tablet H has a double target weight compared to tablet L). They follow the same granulation process and have the same fixed batch size of approx. 250 kg tableting mass. The tablets developed for the two strengths differ in tablet shape (tablet H—oval and tablet L—capsule). The existence of multiple strengths of the same product on the market and the dose-proportionality are defined in the EMA guidelines for process validation [25], bioequivalence [26], or stability studies [27] as a factor to take into consideration in case of bracketing approaches for such studies.

The formulation consists of one active ingredient (>60%; crystalline powder) and well-known excipients fulfilling the roles of binder and diluent (starch and microcrystalline cellulose), surfactant (sodium lauryl sulfate), disintegrant (sodium starch glycolate), glidant (colloidal silicon dioxide), and lubricant (magnesium stearate).

During 18 months of manufacturing, the product presented variable core tablet hardness for both strengths, in the entire range allowed, with some results also below the applicable in-process control range for this parameter. Low hardness values have a negative influence on the appearance of the film-coated tablet due to their increased surface erosion during the coating run. This unexpected variability was observed starting with the second manufacturing campaign. A manufacturing campaign is defined by the EU GMP guideline and is represented by batches manufactured successively on the equipment train in between major cleanings [28]. The grouping of batches in manufacturing campaigns is very relevant for assessing industrial data in the case of frequently manufactured products, like the one included in this study, with more than 50 batches/year, to observe if there are any trends within a campaign or between campaigns.

### 2.2. Process Summary

**Figure 1:**
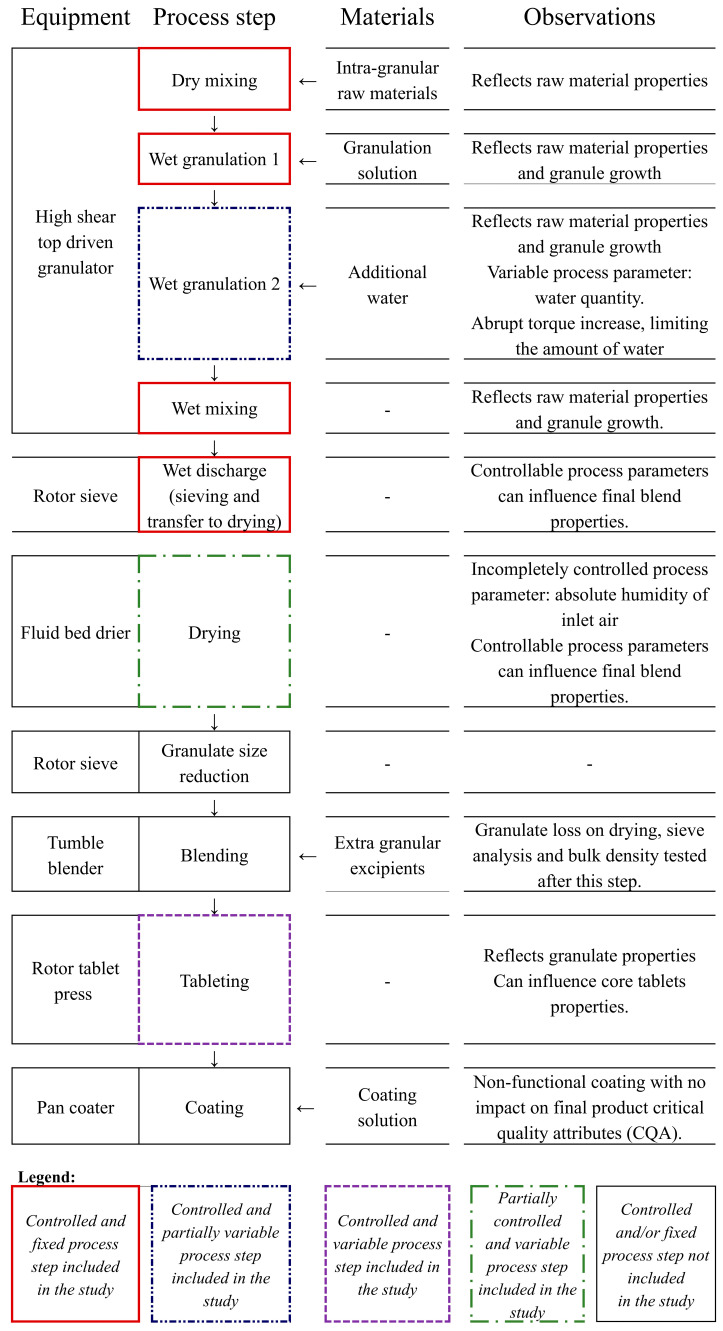
Summary of the manufacturing process.

The manufacturing process involved the following steps with the detailed process flow and equipment types presented in Figure 1: high-shear wet granulation, followed by wet granulate sieving and closed transfer to the fluid bed drying, milling, blending, compression, and film-coating. The steps in scope were fixed, variable, and partially variable with respect to process parameters’ values.

**Figure 2:**
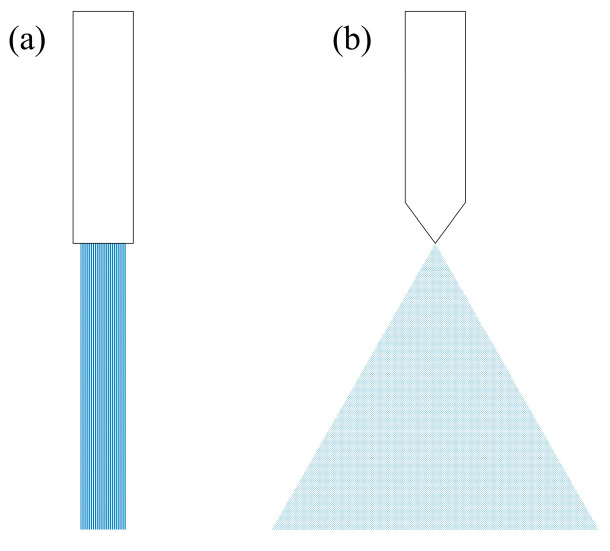
Nozzle types used during high-shear wet granulation ((**a**)—type 1: cylindrical (**b**)—type 2: conical).

Two different types of nozzles (Figure 2) were used for the liquid addition during the high-shear wet granulation steps: type 1 (liquid added in a continuous jet, ensuring a very low contact surface with the powders bed) and type 2 (liquid is dispersed into droplets, ensuring a wider spread of the wetting area). For both nozzles, a peristaltic pump was used as the delivery method, and the same liquid flow rate was set.

The closed transfer of the wet granulate is ensured by the pressure difference created in the fluid bed dryer, which is regulated through the inlet air flap position (% opened) at a constant outlet air flap position (% opened). The less the inlet air flap is opened, the higher the pressure difference and power of suction from the wet mill. The position of the inlet air flap had different set points over certain time intervals during the wet granulate transfer in order to ensure an efficient transfer of the granules between the two pieces of equipment.

Two different tablet press models were used for the batches included in this study. Both were rotary tablet presses, the difference between them being the weight control system at the compression station, in the case of TP1, and at the pre-compression station for TP2, and the number of punches/rotations was 30 and 21, respectively. The dwell times at equal tableting speeds can be considered similar between the two tablet presses (the dwell times of TP2 are 0.7% higher than those of TP1 at the same tableting speed).

Further details regarding composition, raw material suppliers, batch size, and the equipment train could not be disclosed, but there was no difference between batches with respect to these.

### 2.3. Data Collection and Organization

Historical data available from the commercial manufacturing of 114 product batches were compiled (71 for tablet H and 43 for tablet L), belonging to 14 different manufacturing campaigns, and were used for the initial modeling and root cause analysis. The categories of data of interest are presented in Table 1, and the raw data were organized in the proper manner for batch statistical process control [29]. The input variables with the highest variability were selected and the following data tables were generated:

- Batch conditions data (one row per batch): active ingredient batch particle size distribution, granulation water quantity, granulation nozzle type, wet discharge intensity, wet discharge time, drying intensity, inlet air humidity, average tableting speed, average feeder speed, and average compression force.
- Batch evolution data (multiple rows per batch): process variables recorded at regular time points during granulation, wet discharge, and drying.
- Product quality data (one row per batch): granule and tablet quality attributes.

### 2.4. Data Analysis

**Figure 3:**
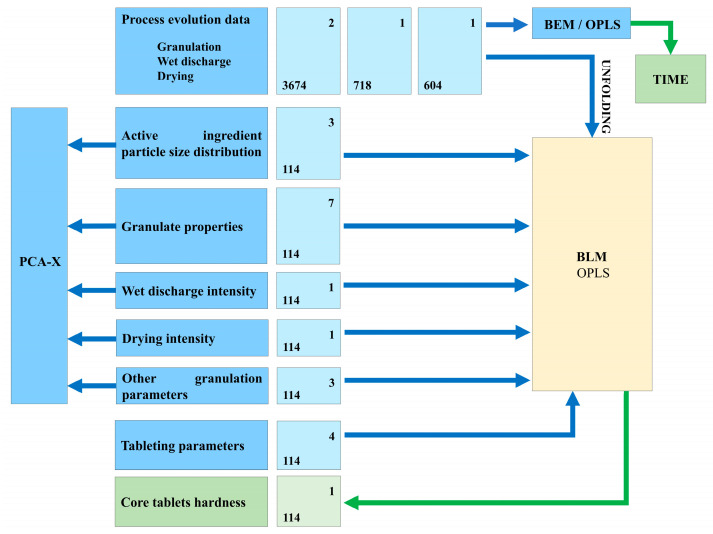
Model building strategy (blue zone—input (X) variables; green zone—output (Y) variables.

Several steps of modeling were performed to detect within-batch variations. The complete overview of the model-building strategy is presented in Figure 3.

Firstly, Batch Evolution Models (BEMs) were built to identify the evolution of time-dependent process variables from the wet granulation, wet discharge, and drying steps. Orthogonal Partial Least Squares (OPLS)-Class models were developed with the recorded process data as an input variable (X) and the time variable as a response (Y). Prior to building the models, the 3-way X matrix (N batches × J time points × K variables) of each process step in scope was unfolded into a 2-way matrix through the combination of batches with time points. The resulting Xu matrix had N × J rows and K columns. Both variables were scaled to unit variance.

Principal Component Analysis (PCA) models were developed to identify different patterns and groups of batches with respect to intermediate product properties or processing conditions. Input data were scaled to unit variance. The model interpretation was performed based on the relevance of each principal component by generating scores and loading plots.

The next step of modeling was the Batch Level Models (BLMs), which were performed in order to compare batches and identify the influence of input data on tablet hardness (Y). These models were developed using the following X dataset: unfolded process data transformed to one row per batch (N rows, K × J columns), active ingredient particle size distribution, intermediate product properties, and additional processing conditions. Prior to fitting the OPLS-Class models for each strength, the variables were scaled to unit variance.

For the visual assessment of the influence of time-dependent process variables on Y, the Sources of Variation Plots (SOV) were used, as they represent the loading of the OPLS model’s predictive component versus time. The impact of other input variables was assessed through the loading plots.

In order to evaluate the performance of the models, either BEM or BLM, the following parameters were considered: the percentage of explained variability in X (R2X) and Y (R2Y), the predictive ability (Q2), and the root mean square error of cross-validation (RMSECV) calculated using partial cross-validation. In order to avoid model over-fitting, the number of latent variables was selected based on the cross-validation criteria. All the models were developed using Simca 17 (Sartorius Stedim Data Analytics AB, Umeå, Sweden).

### 2.5. Effectiveness Check of Improvement Actions

Additional data were collected for 46 batches (32 for tablet H and 14 for tablet L) belonging to five different manufacturing campaigns and manufactured in the next 5 months after the batches included in the initial modeling. These batches were used to assess the effectiveness of the updated control strategy over the API particle size distribution and the granulation process variables which were identified as the main contributors to low hardness values based on the models developed on the initial data set.

The Lean Six Sigma DMAIC methodology was used to classify the different batches included in this study:

1. Define and Measure phases: batches belonging to campaigns from 2 to 5, where the increased hardness variability and tendency for results close to the lower limit and below it was observed; these were the batches that defined the problem statement and triggered the extensive investigation.
2. Analyze and Improve phases: batches belonging to campaigns from 6 to 14, where improvement actions were gradually implemented based on the outcome of the statistical analysis performed.
3. Control phase: additional 46 batches not included in the initial models; part of campaigns from 15 to 19 where all improvement actions were in place.

The sigma quality level (SQL) was calculated for each group using the formula below:

(1) $$SQL=\frac{Min\;[\left(Sample\;Average-LSL\right),\left(USL-Sample\;Average\right)]}{SD},$$

*LSL*—lower specification limit; *USL*—upper specification limit; *SD*—sample standard deviation.

The SQL value can be used to quantify the level of quality of a certain process, with values of 6 corresponding to top performance and with only 3.4 quality deviations per million opportunities [18,30].

## 3. Results and Discussions

The correlation between the evolution of process parameters and product characteristics was assessed through the BLMs generated by regressing the unfolded and rearranged process data matrix and the additional variables against the core tablet hardness values. As the applicable hardness range was different between the two strengths due to tablet size and shape, two separate models were built, one for each strength (Table 2).

For the tablet H batches, the BLM1 showed that 37.9% of the variability in input parameters was correlated with 70.6% of the hardness variation, while, for the tablet L batches, a higher variability in input parameters (59.3%) was correlated with a higher percentage of hardness variation (80.7%), with both models having a decent predictability. The computed RMSECV values are low relative to the typical values of tablet hardness; hence, they confirm the good predictive ability of the models.

### 3.1. Influence of Process Evolution on Core Tablet Hardness

During wet granulation 1, the evolution of the torque measured at the mixer level is an indication of granule growth and consolidation [31]. In the case of this product, based on the visual assessment of the torque profiles generated from BEMs, it was observed that it could be influenced by the nozzle type used for liquid addition and the API particle size. Batches with coarser active ingredient particles generally presented a faster onset of torque increase (after 100–120 s), while, for finer batches, the torque increase occurred at 200–250 s, due most probably to the different liquid-to-solid ratio needed for the powder bed to form agglomerates through coalescence phenomenon until a regular increase in granule size was achieved and the breakage phenomenon began [3,32].

**Figure 4:**
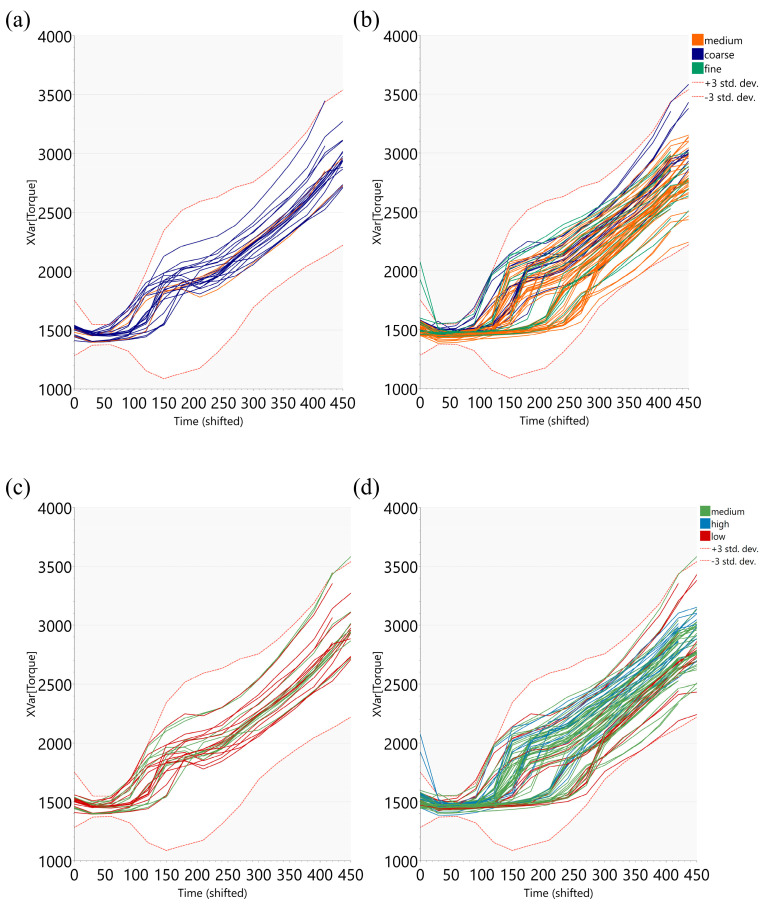
Torque value control chart generated from BEM model for wet granulation 1. (**a**,**c**)—granulations performed with nozzle 1; (**b**,**d**)—granulations performed with nozzle 2. One line represents one batch; batches colored according to API d0.5 classes (**a**,**b**) and according to hardness classes (**c**,**d**).

The batches granulated using nozzle type 1 showed a smoother torque increase compared to the ones granulated with nozzle type 2, within a certain API particle size category (Figure 4a,b). A steep torque increase is an indication of a more intense granulation, with a more efficient granule growing and shaping that leads to higher frictional forces [31,33].

With respect to the output variable in the scope of this study, a steeper torque increase for nozzle 2 seemed favorable for higher hardness values. (Figure 4c,d).

Wet granulation 1 did not capture completely the inter-batch variability in intermediate products, but it suggested that a proper wetting of the API particles had to be ensured in the wet granulation steps with a high focus on coarser API particles having a lower contact surface. This can be achieved by using nozzle type 2, which ensures a dispersion of liquid drops over a larger surface, leading to improved liquid distribution [9,19]. As Figure 4a,c suggested, coarse API particles granulated using nozzle type 1 offered tablets with lower hardness values.

The particle size of raw materials is known to influence granule strength, size, and polydispersity with a consecutive effect on tableting properties. The relationship between the starting size of raw materials—granule properties—and tablet hardness is further influenced by the deformation mechanism involved in tableting. Coarse raw materials usually lead to larger and more uniform granules [5] that have less efficient particle packaging during compression compared to polydisperse samples and often produce compacts with lower solid fractions [34]. A predominant plastic mechanism of particle deformation during compression can further amplify the impact of granule size on tablet hardness, as there is a limited fragmentation phenomenon involved in the formation of efficient bonding surface areas [35].

Tan et al. [36] have investigated the impact of different viscous binder delivery methods (pour-in, pump-in, and spray-in) on granule size, crushing strength, and flowability. The only statistically significant differences were found between the pour-in method and the pump- and spray-in methods due to the uncontrolled liquid delivery rate in the case of the manual pour-in. However, in the current dataset, the additional variability of the API particle size had to be considered, which could affect the dynamics and wettability of the powders, increasing the significance of the liquid addition method. By improving the liquid distribution and keeping the same amount of total granulation liquid, a stronger granulation is ensured for both steady growth and induction growth regime [19,37], which confirms the applicability of nozzle type 2 in the case of both coarse (steady growth regime) and fine (induction growth regime) API.

Other studies have highlighted a larger impact of the binder-addition method for fine raw materials, as small particles require more liquid for agglomeration [5]. Granulation of finer raw materials often leads to the formation of granules with increased strength due to the higher number of bridges between particles with consecutive effects on compressibility during tablet production [38].

In wet granulation 2 and wet mixing steps the torque value generally reached the maximum capacity of the granulator, making more water addition impossible and leading to short process times. For similar end-torque values, variability was observed in the hardness of core tablets; hence, the granulate properties are further influenced by subsequent process steps.

**Figure 5:**
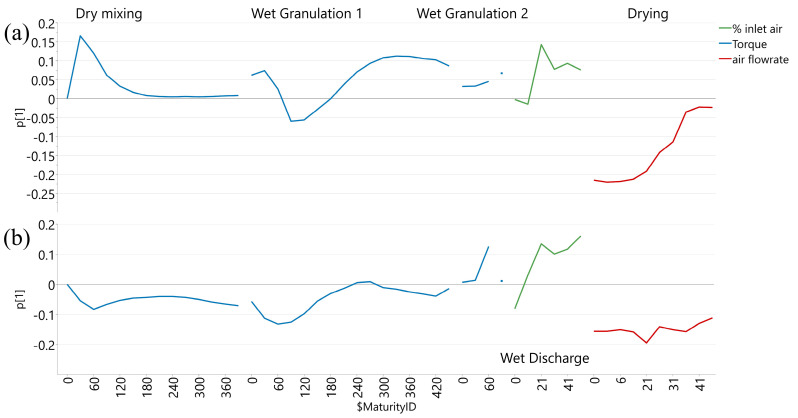
Sources of variation plots for tablet hardness for tablet H (**a**) and tablet L (**b**) batches.

From the SOV plot for these models (Figure 5), it can be interpreted that a steep torque increase occurs between minutes 2 and 5 of wet granulation 1, a visible torque increase in wet granulation 2, a wider opening of inlet air flap during wet discharge (lower transfer intensity), and lower airflow rates at the beginning of the drying step (less intense drying) are correlated with higher hardness. The adjustments made to the input parameters during wet discharge and drying influenced the intensity of the two process steps and, subsequently, the potential mechanical impact on the granules through breakage and attrition. Due to the wet phase of the granules, characterized by weaker bonds, they are sensitive to air friction and inter-particular and equipment collision at a high velocity, especially in the case of formulations without strong binders [39]. Therefore, this influence could be more significant in the case of under-granulated powders obtained by either improper wetting due to coarse particles or insufficient water amount in the case of fine particles.

Among these processes, the variable ones with respect to process parameters are the wet discharge and drying steps. Therefore, by regulating the intensity of these process steps based on the wet granulation torque as an indicator of the granulation strength, the variability of API batches could be leveraged within the registered ranges for water quantity and still yield acceptable tablet hardness values. The actual settings for the intensity of the wet transfer process were also dependent on the density and adherence of the wet granulate; therefore, the minimum intensity settings that ensured adequate throughput without major clogging had to be applied.

Based on this observation, the WD_i_ (wet discharge index—Table 1) and D_i_ (drying index—Table 1), as indices of intensity of these two processes, were calculated from the process evolution data and used further for easier comparison between batches.

All potentially relevant parameters from this assessment were further evaluated in order to understand how different variables can be adjusted inter-dependently in order to improve core tablet hardness values.

### 3.2. Influences of Active Ingredient, Granulation Conditions, and Intermediate Product Properties on Core Tablet Hardness

**Figure 6:**
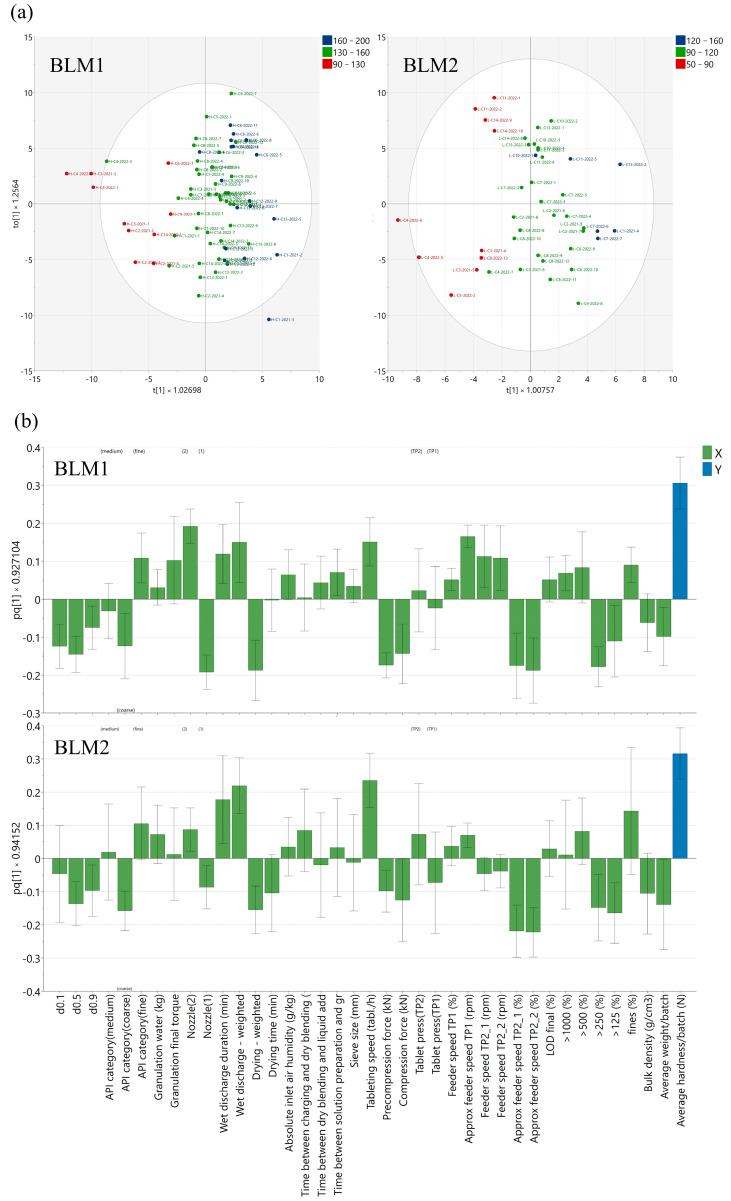
Score plots (**a**) and corresponding predictive loadings (**b**) of BLM1 and BLM2.

The score plots generated from BLM1 and BLM 2 (Figure 6a) show that, by grouping the batches in hardness classes considering the specification ranges of low (lower third), medium (middle third), and high (upper third), the batches are relatively well separated; therefore, adequate processing conditions could be identified. Batches from the beginning of one manufacturing campaign generally presented lower hardness values (*p* = 1.15 × 10^−5^).

The predictive loading plots generated from BLM1 and BLM2 (Figure 6b) were further assessed, and the correlations observed for the computed wet discharge intensity index (positive loading) and drying index (negative loading) confirm the observations from the SOV plots, having an important contribution to the statistical significance of the model.

Related to the wet granulation process conditions, the nozzle type had an impact on hardness: nozzle type 2 (positive loading) and nozzle type 1 (negative loading).

The granule properties with the highest inter-batch variability showed a good correlation to the average hardness/batch: fractions >250 and >125 microns (negative loadings for both strengths) and fines (positive loading for tablet H). This is in line with the expected influence of granulate particle size on tabletability [40,41], as a higher fraction of large particles can reduce the deformation potential of the granules, leading to a lower tablet hardness. Macho et al. highlighted a similar relationship between the granule particle size and tablet tensile strength for high API-loaded products. Higher tensile strengths could be obtained from smaller granules presenting a lower conditioned bulk density [42].

The inverse correlation between torque and granulate particle size is atypical, as, normally, a more intense granulation (higher torque) would imply higher granulate particle sizes. This could be explained through the impact of reducing breakage and the attrition of the granules through better distribution of the granulation liquid [9,19] and less intense wet discharge and drying conditions, which prevent larger granules from breaking up into intermediate-sized granules [39] (nozzle type, wet discharge, and drying index as confounding variables).

The API particle size distribution is also a main contributor to the model. Lower particle sizes are preferable for optimizing tablet hardness; however, variability is observed between batches having similar API particle sizes. The favorable impact of lower API particle sizes could be explained by the higher interparticulate bonding surface area, which leads to the formation of granules with improved compressibility [5,34,43].

### 3.3. Influences of Tableting Process on Core Tablet Hardness

From the loading plots (Figure 6b), it is visible that higher tableting speeds (positive loafing) and lower compression forces (negative loading) are correlated with higher hardness values. This is explained by the fact that the majority of batches leading to adequate hardness values upon tableting (medium and high hardness classes) could be processed at speeds in the upper half of the product-specific range (IQR = 44–56 k tablets/hour for tablet H and IQR = 57–67 k tablets/hour for tablet L) without excessively increasing the tableting force (IQR = 24–31 kN for tablet H and IQR = 25–30 kN for tablet L) due to the adequate compressibility of these blends. Applying the same tableting conditions on blends with poor tableting properties led to borderline or even out-of-specification hardness. Thus, for these blends, for which a hardness plateau was reached at low values (lower third of the specification limit or below), a lower tableting speed (from 20 to 30% decrease) and higher compression force (from 10 to 15% increase) was used to maximize hardness by increasing the dwell time. When high forces are needed to reach the desired hardness, the granulates densify mainly by particle deformation [44], thereby also triggering a higher elastic recovery, which results in poor tablet mechanical resistance [45] and reaches a plateau due to the leveling produced by the elastic recovery [46].

Therefore, by obtaining granulates that can be processed at optimal hardness values, tableting throughput can also be increased, which is a desired additional improvement.

The feeder speed of TP1 was also positively correlated with the tablet hardness values, but this was also dependent on the tableting speed value. In the case of TP2, the feeder speed in rpm shows a similar correlation as in the case of TP1. However, an inverse correlation is seen if the feeder speed is expressed as a percentage of the tableting speed, suggesting a potential improvement opportunity in the tableting step, through reduction of the relative feeder speed to avoid any potential impact of over-lubrication or increased granule attrition caused by increased shear in the feed frame [47,48,49].

The difference between the two tablet presses was further investigated for both strengths by building OPLS-Class models (BLMs 3–6). All models obtained have decent predictability (Table 2) and were built in order to better understand if there are any significant differences between the two tablet presses with respect to product performance.

The impact of the relative feeder speed in the case of TP2 showed a similar negative loading for both tablet types. The wet discharge index has a similar positive loading for all groups. The impact of the nozzle type and drying index is more clearly visible for tablet H batches processed using TP2, as most batches with nozzle 1 and a high drying index were processed with this tablet press. For the same strength, the impact of the wet discharge index is more visible for TP1, as, in this case, the loading of the other two main variables was partially leveled. The drying index is statistically significant only for batches processed using TP2. The API particle sizes showed negative loadings in the case of tablet L batches processed on TP2 and tablet H batches processed with both tablet presses, although a similar inter-batch variability is observed in all groups (Figure S1).

The comparison between the two tablet presses, using this data set, suggests that API particle size and granulation conditions can have a greater impact on the tablet hardness of both tablet types processed using TP2 and tablet H batch processes using TP1. Tablet L batches processed with TP1 show a positive loading only for the wet discharge index and time. The hardness variability between the groups is similar; hence, no significant difference can be identified between the two tablet presses (Figure S2), just additional hardness fine-tuning opportunities.

The different granulate properties, influenced by the granulation conditions stated above, can lead to different behaviors in the tableting process without multiple adjustments available in this process unit. Loss of compatibility for certain granulates can lead to a larger part of the applied compression force during tableting being used to break up the granules, reducing the strength of the inter-particulate bonds [50].

### 3.4. In-Depth Analysis of Batch Groups Based on Most Significant Process Conditions

**Figure 7:**
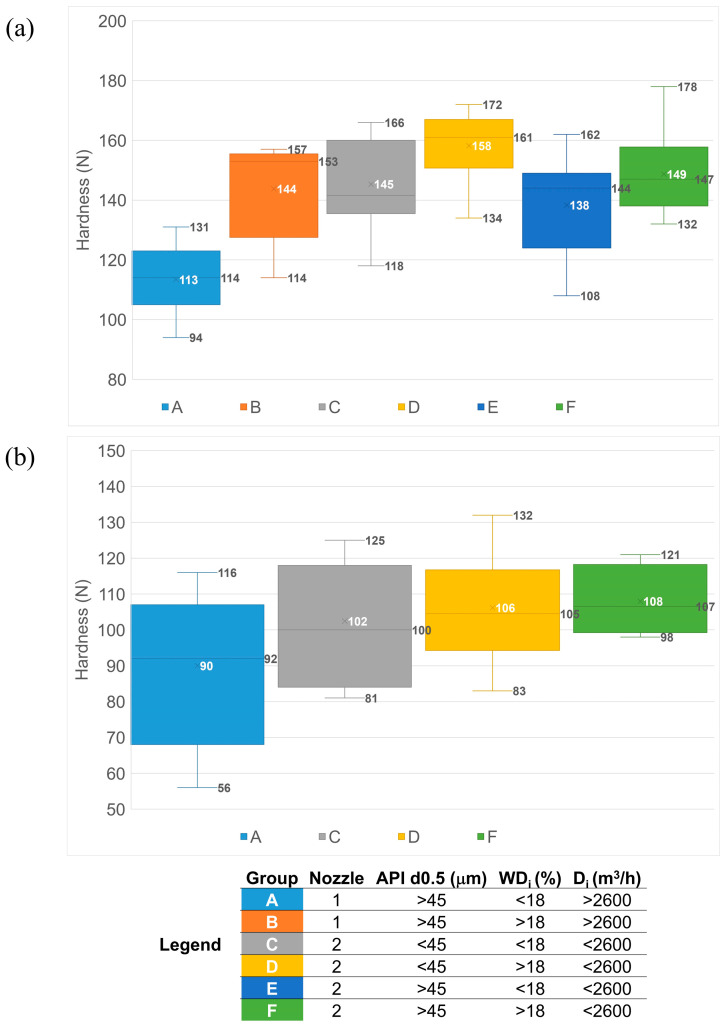
Hardness comparison for tablet H (**a**) and tablet L (**b**) batches process condition groups.

The batches were split into several groups based on the most important variables identified in the BLM models (nozzle type, API d0.5, WD_i_, and D_i_) and were compared regarding the average core tablet hardness and variability within the groups. Statistically significant differences were found between some groups of tablet H (*p* ANOVA—1.91 × 10^−8^, Figure 7a), while, for tablet L, the average hardness value was similar between the groups (*p* ANOVA—0.16, Figure 7b).

The lowest hardness values for both tablet types were obtained for batches granulated with nozzle 1, having API d0.5 > 45 microns, a wet discharge index below 18% (intense transfer), and a drying index above 2600 m^3^/h (intense drying). These working conditions were applied mainly for batches belonging to manufacturing campaigns from 2 to 5. Starting with campaign 5, only nozzle 2 was used. Starting with the same campaign, the drying intensity was reduced; therefore, in the groups generated using nozzle 2, all batches have a drying index below 2600 m^3^/h. Therefore, starting with campaign no. 6, the process was partially improved and was under further assessment.

As more data were available for the tablet H batches, a detailed analysis of all the groups was possible only for this tablet type. When comparing the groups of batches granulated with nozzle type 2, the highest average hardness/group and lowest variability within the group were observed for batches using API with d0.5 < 45 microns and a wet discharge index above 18%. Therefore, in addition to the optimized drying conditions, these values of the other controllable input parameters could be considered as a reference (Figure 7a). A wet discharge index above 18% would, therefore, be recommended regardless of the API particle size distribution, with a statistically significant difference for the average hardness value of group D and a reduction in within-group variability in the case of group F.

In the case of the tablet L batches, there is a higher scattering of the results within each group, leading to inconclusive statistical evaluation; however, the variability within the groups C, D, and F (API d0.5 < 45 and/or wet discharge index > 18%) is the lowest among all groups, suggesting a more robust process (Figure 7b). The inconclusive statistics in the case of tablet L could be driven by the smaller data set that was available for evaluation and the fact that there were not batches for all the groups defined. Also, the impact of the granulate properties on the mechanical resistance of the tablets is influenced by their shape and size, with the mechanical activation being higher in smaller tablets [51], making the smaller and capsule-shaped tablets less sensitive compared to the larger and oval-shaped tablets, considering the final blend is identical. The geometry of the tablets influences the distribution of the compression force during the tableting process, leading to non-uniform density regions within the tablets, which trigger the hardness variability; convex tablets are more sensitive compared to flat-faced tablets [52].

The major impact of the above-mentioned parameters (API d0.5, nozzle type, wet discharge, and drying intensity) is also demonstrated by the clear difference between batches shown by the score plot of a PCA model built using all the input parameters for both strengths of the product. The model was fitted with four principal components, explaining 58.9% of the variability in the investigated characteristics. Batches belonging to groups A and B are clearly separated from the other groups, being characterized by the usage of nozzle 1, a higher drying intensity index and a lower wet discharge index, higher API particle sizes, and higher fractions of granules above 250 and 125 microns (Figure S3).

### 3.5. Long-Term Effectiveness of the Proposed Control Strategy

All the objectives of a Lean Six Sigma methodology [17] were reached through the updated control strategy:

- Waste reduction:
  ○ Fewer product losses during the prolonged set-up needed for lower hardness batches.
  ○ Significantly reduced risk of eroded tablets after film-coating, leading to visual sorting for the removal of defective tablets
  ○ Significantly reduced risk of broken tablets during packaging caused by the low hardness
- Reduction in inter-batch variability through defining more standardized processing conditions.
- Process efficiency improvement through increased tableting throughput, as batches with medium and high hardness can be processed at a higher tableting speed.
- Improved product quality at core tablets in-process control level through a reduction in the deviation rate for hardness values below the acceptable limit.
- Cost reduction through a reduction in overall processing and batch release times.

**Figure 8:**
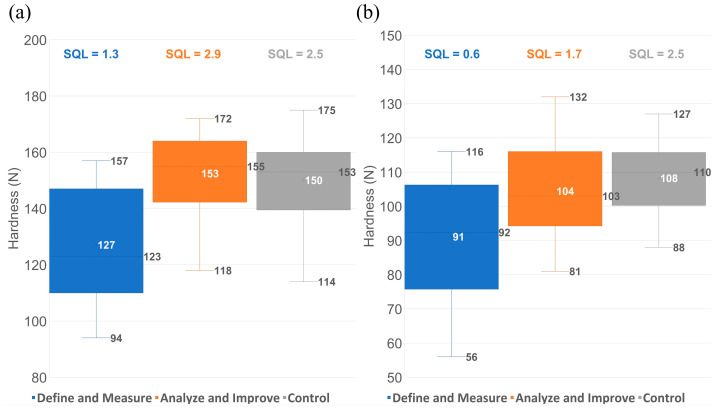
Hardness and sigma level values for tablet H (**a**) and tablet L (**b**) batches before and after new control strategy implementation.

For the numerical assessment of the improvement, the average hardness, inter-batch variability, and sigma quality level were calculated for each group listed in Section 2.4 (1—Define and Measure phase; 2—Analyze and Improve phase; 3—Control phase) and were compared. There was an average hardness increase of 18–20% in the case of the tablet H batches and 15–20% for the tablet L batches processed in ideal conditions, representing a significant increase and, at the same time, showing a lower inter-batch variability. The performance of the process in the control phase (III) versus the improve phase (II) decreased slightly but was still within the 1.5 sigma shift in the case of tablet H, while, for tablet L, the performance improved further (Figure 8).

The 1.5 sigma shift is an expected process control drift over time [30] due to the combination of common and special cause variation on long-term assessment. Both strengths reached the same sigma level in the control stage (SQL = 2.5), a value that is currently typical for the pharmaceutical industry [18]. This proves that there is an increase in process robustness when applying a more standardized process, which could open the opportunity for further improvement.

## 4. Conclusions

The purpose of this study was to assess the impact of process evolution, apply a systematic root cause investigation for low and variable core tablet hardness using production run data recorded over 18 months, and assess the effectiveness of the applied improvement actions on newly manufactured batches. For this purpose, the multivariate data analysis tools managed to integrate the process evolution data with the particle size of the active ingredient, properties of the intermediate product, and different processing conditions to identify four main variables affecting the output variable in scope: API particle size, nozzle type used for liquid addition in the granulation phase, wet discharge, and drying intensity.

The BEMs revealed the desired pattern of process evolution for reaching medium and high hardness values, facilitating the identification of flexible variables that can be adjusted in order to optimize the process pattern.

Through BLMs and BLM-class models, ideal processing conditions could be defined, leading to a new control strategy with at least three out of the four variables affecting hardness having to be within the desired ranges. In the case of this product, the desired API particle size distribution should present a d0.5 below 45 microns; however, newly defined processing conditions (nozzle type 2, wet discharge index above 18%, and drying index below 2600 m^3^/h) could accommodate values of up to 70 microns, based on the existing data-set, and still ensure adequate core tablets hardness (at least 30% above the lower specification limit) for the successive film-coating step. This means that the manufacturing process can leverage API variability while ensuring both product quality and production efficacy (tableting speeds in the upper quartiles of the historical product-specific dataset) without the need to implement very stringent specification limits and process parameter ranges, which could be prone to frequent deviations.

The effectiveness of the new control strategy was confirmed through the significant average hardness increase (15–20%) and between-batch variability decrease, leading to decent sigma quality levels for the control phase batches (SQL = 2.5).

Through this modeling exercise and systematic root cause investigation, methodology product and process knowledge was increased, and the usability of multivariate analysis tools was confirmed once again for industrial data sets and tablet manufacturing, despite data assessment and interpretation having to be performed differently from pre-defined designs of experiments due to the inter-related variables arising from non-simulated conditions. Although the optimal processing conditions as absolute values defined in this study are product-specific, the detailed step-wise methodology described in this study can be applied to any similar product and process to identify process improvement opportunities using historical datasets.

## Figures and Tables

**Table 1:** Data collection plan.

Variable Type	Attribute (Unit of Measure)		Data Source
Raw material	API	Particle size distribution (PSD) by laser diffraction (d0.1; d0.5; d0.9) (μm)	Certificate of analysis; one value/batch
Process parameters	Wet granulation	Mixer speed (rpm)	Equipment report; Datapoints at 30 s interval
		Torque value (Nm)	
		Quantity of granulation water (kg)	Batch records
		Nozzle used for liquid addition (type 1 and type 2—Figure 1)	Batch records
	Wet granulate discharge	Inlet air flap position (%)	Equipment report; set point per time interval
		Wet discharge intensity index (%; WD_i_) (low values represent high intensity)	Computed from above variable as weighted average of the various the set-points during a batch with their corresponding durations
		Wet discharge time (min)	Equipment report
Process parameters	Granule drying	Inlet air flowrate (m^3^/h)	Equipment report; rounded actual value per time interval
		Drying intensity index (m^3^/h; D_i_) (low values represent low intensity)	Computed from above variable as weighted average of the various the set-points during a batch with their corresponding durations
		Inlet air humidity (g/kg)	Equipment report
		Drying time (min)	Equipment report
	Tableting	Tablet press model (TP1/TP2)	Batch records
		Speed (tablets/hour)	Batch records; data points at 1 h interval
		Feeder speed (rpm or %)	
		Pre-compression force (kN)	
		Compression force (kN)	
Granule properties	Final blend	Particle size analysis by sieving (% retained on 1000; 500; 250; 125 microns sieves and % of fines below 125 microns)	Batch records
		Bulk density (g/mL)	
		Loss on drying (LOD) (%)	
Core tablet properties	Average hardness/batch (N)		

**Table 2:** Performance parameters of first-phase BLM models fitted using PLS and second-phase OPLS-Class models.

Nr.	Y	X	R2X (cum)	R2Y (cum)	Q2 (cum)	RMSECV
BLM1	Tablet hardness (tablet H)	API particle size distribution Granulation pp Water quantity Wet discharge pp Drying pp Granule properties Tableting pp	0.379	0.706	0.646	11.162
BLM2	Tablet hardness (tablet L)		0.593	0.807	0.665	9.827
BLM3 TP1-H	Tablet hardness	Granulation conditions Granulation final torque Water quantity Absolute inlet air humidity (drying) Sieve size Granule properties Tablet press type Tableting feeder speeds (%)	0.644	0.835	0.635	10.203
BLM4 TP2-H			0.446	0.765	0.678	10.572
BLM5 TP1-L			0.945	0.999	0.841	4.996
BLM6 TP2-L			0.448	0.814	0.682	9.994

pp—process parameters; TP1/2—tablet press.

## Data Availability

The original contributions presented in this study are included in the article/Supplementary Materials. Further inquiries can be directed to the corresponding author.

## Supplementary Materials

The following supporting information can be downloaded at https://www.mdpi.com/article/10.3390/pharmaceutics17020213/s1: Figure S1: Predictive loading of BLM 3 (a), BLM 4 (b), BLM 5 (c), and BLM 6 (d) models, fitted using PLS; Figure S2: Hardness comparison between tablet presses for both strengths (H—tablet H and L—tablet L); Figure S3: Score and loading plot of PCA model built on process conditions and granule properties. Batches are colored according to groups A to F.

## Abbreviations

API—Active Pharmaceutical Ingredient; BEM—Batch Evolution Model; BLM—Batch Level Model; DMAIC—Define, Measure, Analysis, Improve, Control; EMA—European Medicines Agency; EU GMP—EudraLex—Volume 4—Good Manufacturing Practice (GMP); IPC—In-Process Control; IQR—Inter Quartile Range; LOD—Loss on Drying; PCA—Principal Component Analysis; PLS—Partial Least Squares; PSD—Particle Size Distribution; RMSECV—Root Mean Square Error of Cross-Validation; SD—Standard Deviation; SOV—Source of Variation Plots; SQL—Sigma Quality Level; TP—Tablet Press.
