# Supplementary material for: Multivariate Data Analysis to Assess Process Evolution and Systematic Root Causes Investigation in Tablet Manufacturing at an Industrial Scale—A Case Study Focused on Improving Tablet Hardness
Source: Pharmaceutics. 2025 Feb 7;17(2):213. doi: 10.3390/pharmaceutics17020213 (PMC11858851; doi:10.3390/pharmaceutics17020213)
Supplement: Supplementary file 1 [file pharmaceutics-17-00213-s001.zip › pharmaceutics-3417274-supplementary.pdf]

Supplementary Materials

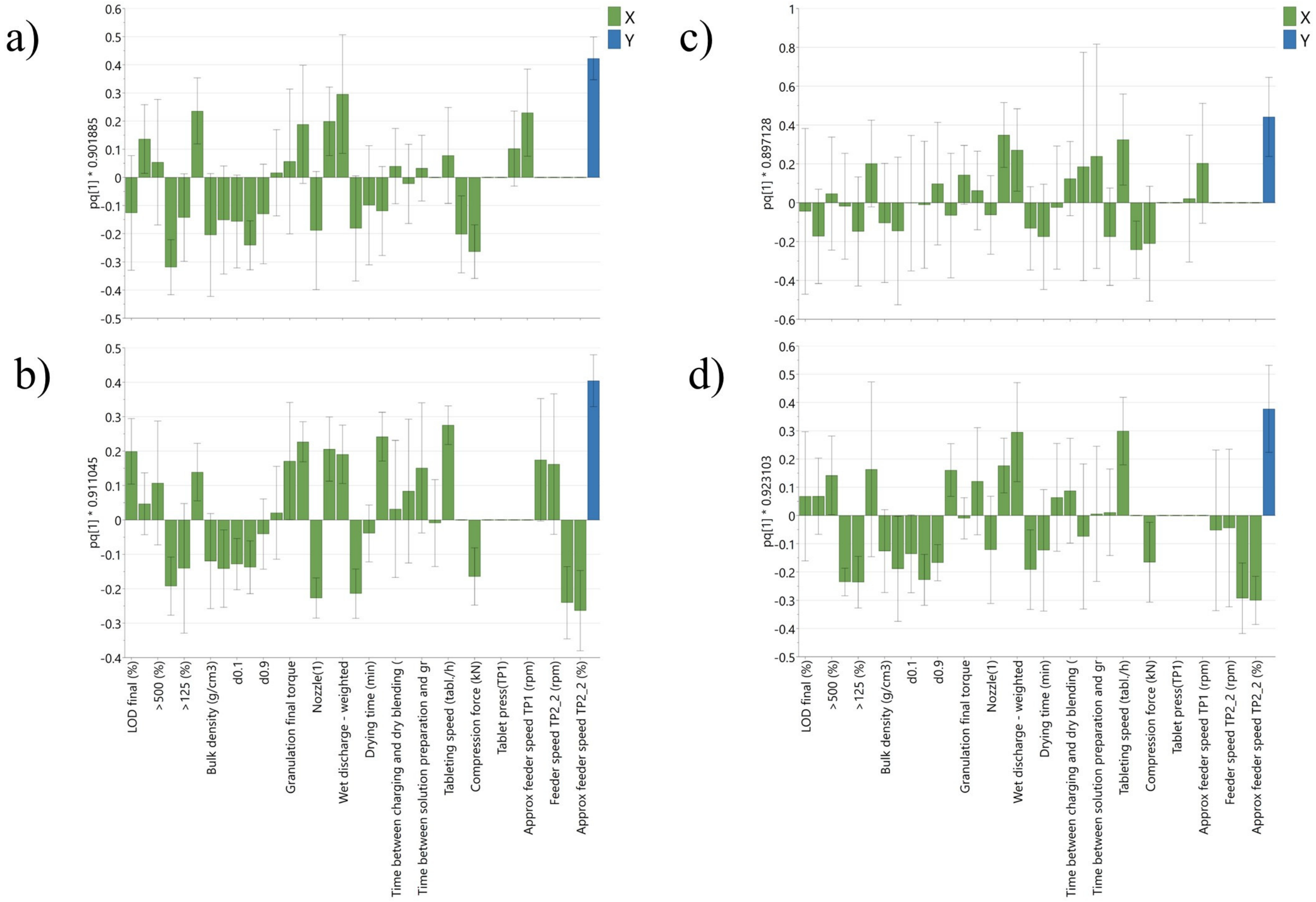

Figure S1. Predictive loading of BLM 3 (a), BLM 4 (b), BLM 5 (c), and BLM 6 (d) models, fitted using PLS.

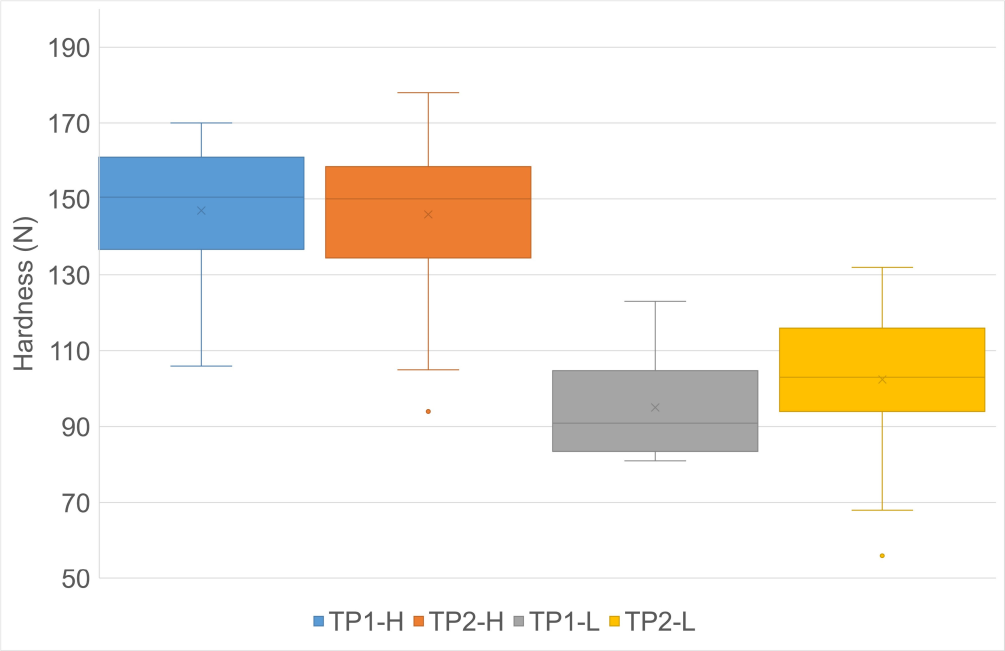

Figure S2. Hardness comparison between tablet presses for both strengths (H—tablet H and L—tablet L).

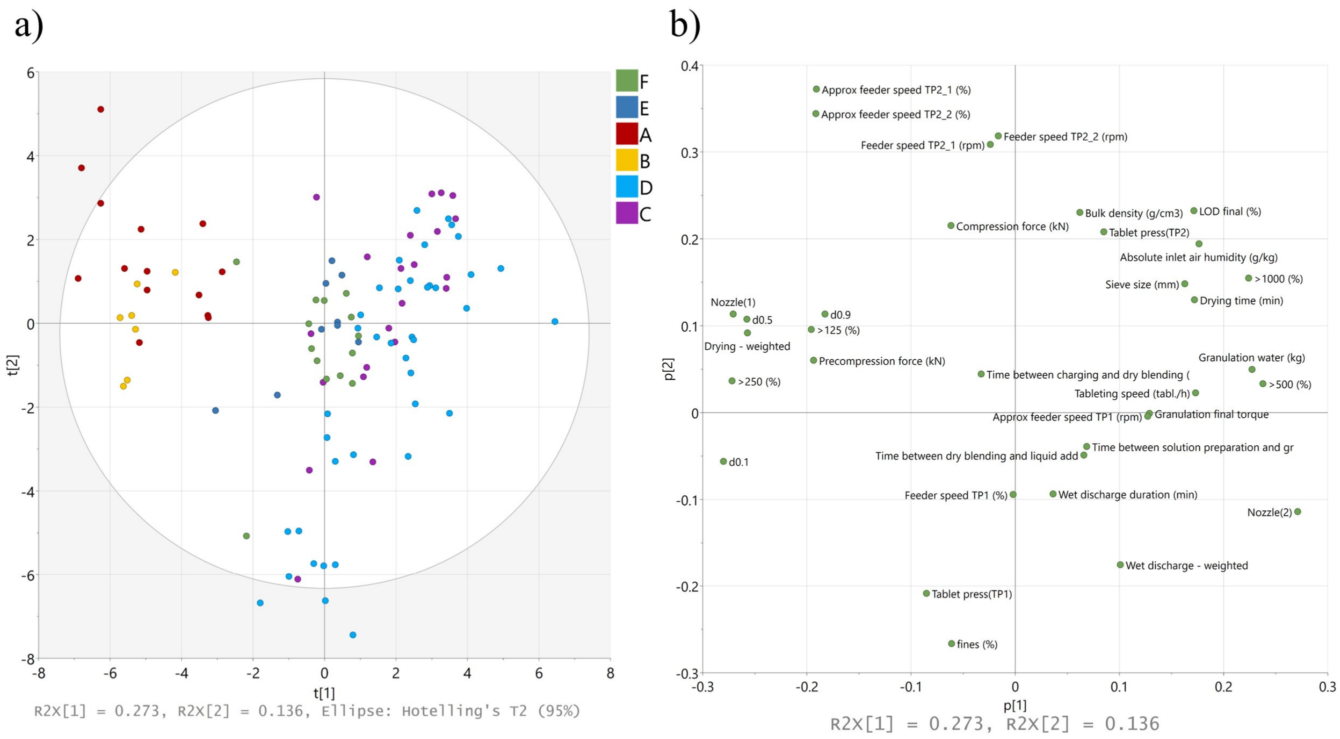

Figure S3. Score and loading plot of PCA model built on process conditions and granule properties. Batches are colored according to groups A to F.
